# The Impact of Melatonin in Research

**DOI:** 10.3390/molecules21020240

**Published:** 2016-02-20

**Authors:** Elena Maria Varoni, Clelia Soru, Roberta Pluchino, Chiara Intra, Marcello Iriti

**Affiliations:** 1 Department of Biomedical, Surgery and Dental Sciences, Milan State University, Milan 20142, Italy; elena.varoni@unimi.it (E.M.V.); clelia.soru@gmail.com (C.S.); pluchinoroberta@gmail.com (R.P.); chiara.intra@libero.it (C.I.); 2 Department of Agricultural and Environmental Sciences, Milan State University, Milan 20133, Italy

**Keywords:** bibliometrics, biological clock, circadian rhythms, endocrinology, sleep

## Abstract

Citation indexes represent helpful tools for evaluating the impact of articles on research. The aim of this study was to obtain the top-100 ranking of the most cited papers on melatonin, a relevant neurohormone mainly involved in phase-adjusting the biological clock and with certain sleep-promoting capability. An article search was carried out on the Institute for Scientific Information (ISI) Web of Science platform. Numbers of citations, names of authors, journals and their 2014-impact factor, year of publication, and experimental designs of studies were recorded. The ranking of the 100-most cited articles on melatonin research (up to February 2016) revealed a citation range from 1623 to 310. Narrative reviews/expert opinions were the most frequently cited articles, while the main research topics were oxidative stress, sleep physiology, reproduction, circadian rhythms and melatonin receptors. This study represents the first detailed analysis of the 100 top-cited articles published in the field of melatonin research, showing its impact and relevance in the biomedical field.

## 1. Introduction

By simply typing the word “melatonin” on the PubMed database, more than 1000 records can be easily retrieved, for just year 2015. Melatonin, the main sleep-promoting neurohormone involved in phase-adjusting the circadian clockworks upon prior phase-shifting, recently received overpowering attention in science, medicine and social media, and it is expected to gain even more attention within the near future.

Such a feeling is strongly supported by analyzing the “citation index” of this molecule, i.e. how many times researchers have cited papers on melatonin over time. “Citation index” is, to date, one of the most reliable methods for assessing the quality and the “scientific power” of a paper, a journal or an issue [1], reflecting its impact on research, opening further discussion, producing changes in clinical practice, starting controversy inside scientific community and providing new perspectives in science and in financial funding as well.

The top-100 rank of the highly cited papers provides an interesting picture of the current “hot” topics, even delineating those trends expected to further explode in the future. Along this direction, the rankings of the 100 top-cited articles have been published in a plethora of biomedical disciplines, such as emergency medicine [2], cardiology [3], orthopedic surgery [4] and dentistry [5]. Considering the wide and increasing interest on this hormone, this work aims to provide and analyze the ranking of the 100 top-cited articles on melatonin research.

## 2. Results

During our search we excluded only one article for being out-of-topic, namely a review by Del Rio *et al*. on the toxic molecule malondialdehyde as a biological marker of oxidative stress [6]. We found that the number of citations in the top-100 rank (Table 1), ranged between 1623 and 310; each of the first five articles exceeded 1000 citations and the first sixty articles had more than 400 citations. These findings provide the major, pivotal hint of the huge impact of melatonin in science, since all papers of our ranking had more than 100 citations, the latter considered the threshold to identify a “classic” article [7,8]—the “last” article, at position 100, had 310 citations. This also suggests that due to the nature of this ranking, a very large number of classics have not been here included, despite their undeniable scientific importance.

The first paper, with 1623 citations, presented a narrative review which included melatonin in the response to oxidative [9]. This was also the first article in the ranking based on the annual citation rate (ACR), *i.e.*, the ratio between the number of citations of a paper and the number of years since its publication: Valko *et al*. collected 1490 citations in 10 years with an ACR of 162.3 (Table 2). The ACR classification also highlighted the work by Galano and colleagues [80] which recorded a very high ACR (=99.7), because their paper, just published in 2011, collected 356 citations.

In second place of the top 100 list rank one could find another narrative review by Reiter and colleagues, published in 1991 [10]. The work entitled “*Melatonin: a potent, endogenous hydroxyl radical scavenger*”, with 1420 citations, completed the podium [109]: This was an original article, published by Tan *et al*., in 1993. The fourth paper , with 1,219 citations [109] was a narrative review published by Reiter *et al*., in 1980. The top 5 ranking concluded with a paper by Lewy and colleagues published in *Science* in 1980, with 1105 citations [13].

**Figure 1:**
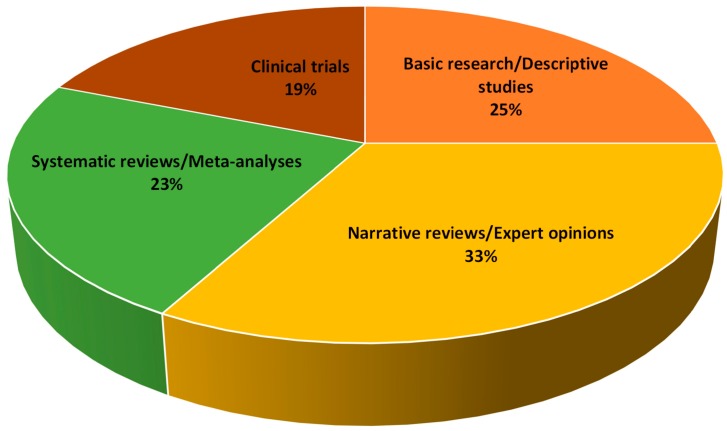
Methodological designs of the top 100 most cited papers in melatonin research.

Unexpectedly, scientific works with limited evidence were cited the most. For the most part, indeed, articles were narrative reviews/expert opinions (33%), followed by basic research/descriptive studies (25%), whilst the less represented papers were systematic reviews (23%) and clinical trials (19%) (Figure 1).

Because reviews are usually more frequently cited, two different top 10 rankings were created to minimize this bias, in order to evaluate in details the number of citations for reviews *vs.* original articles (Table 3 and Table 4). We included one letter to the editor among the original articles [35], since it reported a novel non-extraction radioimmunoassay (RIA) to detect melatonin in plasma. Interestingly, in both the classifications, melatonin as antioxidant agent and its role in physiology, mainly in regulating mammal reproduction, were the most cited topics.

**Figure 2:**
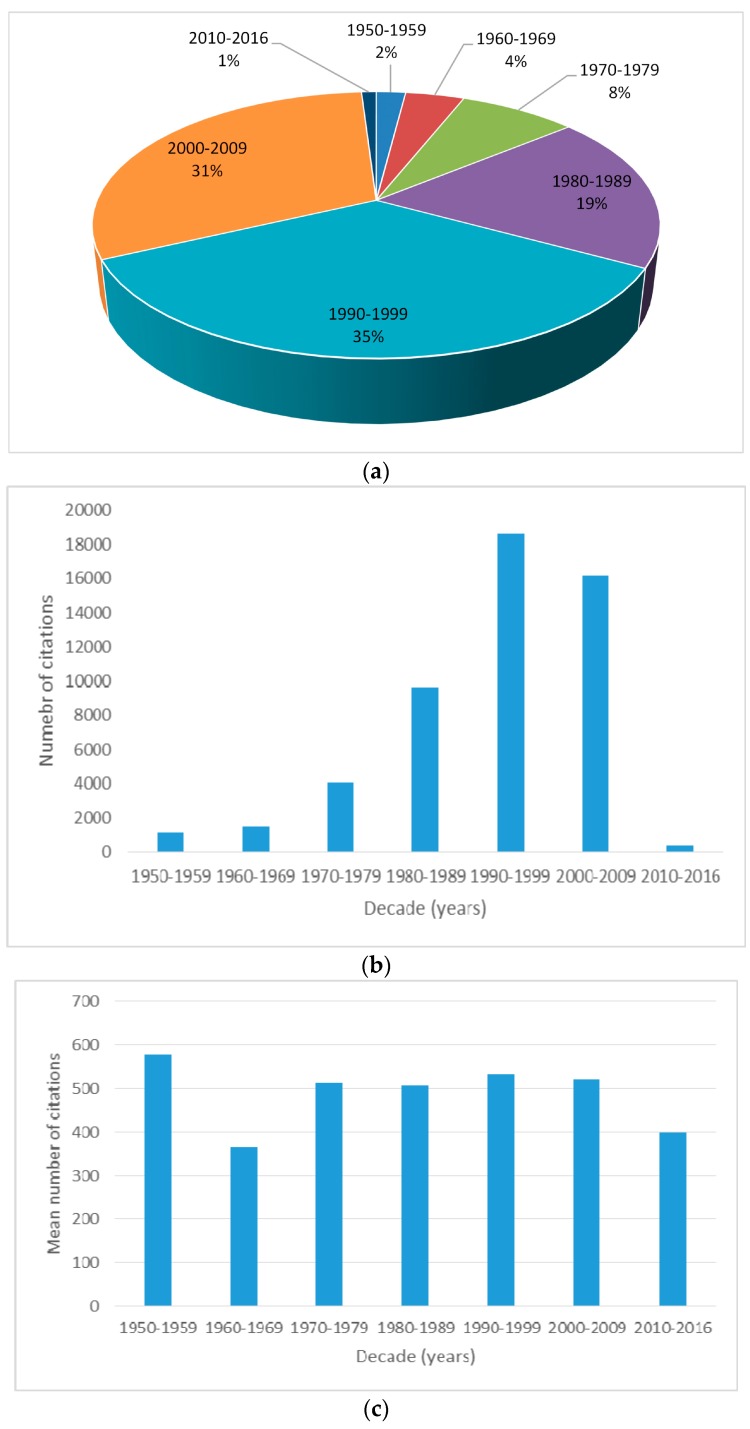
Years of publication (**a**) of the top 100 most cited articles in the field of melatonin research. Number of citations (**b**) and mean of the number of citations per decade (**c**).

The *golden age* for melatonin research, accounting for the largest number of “most-cited” publications, was the 1990–1999 decade, with 35 articles (Figure 2a). This decade also showed the highest number of total citations (18,604, Figure 2b). The 2000s followed with 31 papers and 16,182 total citations. The highest mean of the number of citations, calculated as the total citations from the total number of top-100 articles per decade, was, instead, recorded for the decade of the 1950s, with 578 mean citations (Figure 2b).

The top 100 most cited articles were published in 52 different journals (Table 5). The journal with the largest number of papers was *Science*, with 13 articles, four of them within the first 20. It was followed by the *Journal of Pineal Research* and *Endocrinology*, with eight and five papers, respectively.

**Figure 3:**
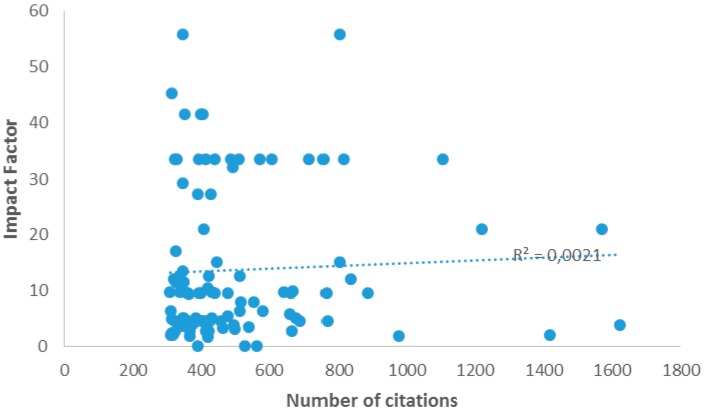
Linear correlation between number of citations of articles included in the top 100 list and impact factors of journals where papers were published.

Surprisingly, no correlation could be observed between the number of citations in this ranking and the impact factors of the journals where papers were published (linear regression: R^2^ = 0.0021, Figure 3).

The authors with the highest number of articles within the rank were Reiter with 16 papers (first author in nine of them), followed by Tan with nine papers (four as first author) and Reppert with six articles (five as first author) (Table 6). At fourth place, Weaver had five papers and was first Author in one of them. At fifth place, Axelrod, Nobel Prize in Physiology or Medicine in 1970, had five article and he was first author in two of them. Axelrod was *ex equo* with Manchester.

## 3. Discussion

Since the first bibliometrics study on melatonin published two decades ago [110], melatonin has acquired more and more the role of a pleiotropic molecule, regulating each aspect of the biological clock, from sleep to appetite and reproduction. The great impact of this molecule on research is reflected by the highest number of citations corresponding to the 1990s and 2000s. Accordingly, the most frequent topics, found in the top-100 ranking, included sleep physiology, reproduction, circadian rhythms, and oxidative stress. These trends were also reflected by the content of the first ten most cited papers. Nonetheless, melatonin research, to date, covers a number of additional fields, besides the biomedical ones, which are expected to greatly contribute to the further importance of this molecule within the next years. Recently, melatonin has become a relevant issue in plant and food sciences [111,112], but we could not retrieve any specific article among the top 100 rank.

Like any other bibliometric study, our analysis is not exempt from a number of limitations. We are aware that other citation impact measures, not included in our analysis, also exist, such as the h-index, and also we did not control for the effects of self-citation. Additionally, in some cases, the number of citations cannot quantify the value of a work contribution to the field [113,114], since this is affected by many bias, mainly temporal ones [115]. Indeed, a paper tends to accumulate citations over time, while recent articles may not have had enough “publication time” to produce high rates in the citation analysis. Conversely, the number of citations may then fall progressively as the content of the paper is absorbed into the current knowledge. Moreover, our methodology was based on the Web of Knowledge platform, referring to all subscribed databases simultaneously consulted for the most comprehensive results. The Web of Science, however, does not index all peer-reviewed journals, thus we might have missed other journals indexed in other databases, such as Scopus. We did not use Google Scholar for this citation analysis, since despite being useful to cover some social and humanities sciences, is not accurate for the biomedical area. It has no quality control, searching within the web for scholarly content and considering, among the others, non peer-reviewed journals, books and academic theses, as well as non-scientific sites, such as promotional ones. The Google Scholar citation index is, thus, not considered highly reliable, at times pre-dating the publication it claims to cite and displaying manifold versions of the same publication, splitting the citation count [116].

## 4. Materials and Methods

In February 2016, we consulted Science Citation Index Expanded™, a specific online resource to quantify citations belonging to the Institute for Scientific Information (ISI) Web of Science™ platform [5]. Under “Basic Research” tag, the keyword (“topic”) used for search was “melatonin” and all the results were sorted using “time cited - highest to lowest”. A second search was then performed under the “Cited Reference Search” tag, using the word “melatonin” as “cited title”: every record were checked to identify the most cited ones and matched with the previously obtained list. The number of citations corresponded to the “Citing Article Counts”, which referred to all databases and all years, *i.e.*, Web of Science™ Core Collection (1985-present), CABI, CAB Abstracts^®^ (1973-present), Inspec^®^ (1969-present), KCI-Korean Journal Database (1980-present), MEDLINE^®^ (1950-present), SciELO Citation Index (1997-present). We deliberately excluded Google Scholar since it is not purposely intended to retrieve citations in a systematic and controlled way, as Web of Science or Scopus do [116]. The resulting 100 most cited articles were selected and full-text retrieved to verify the coherence with the topic (melatonin in research). The following data were recorded for each one: ranking based on the number of citations; number and names of the authors; year of publication; journal in which published and the corresponding 2013–2014 Journal Citation Report - Science Edition impact factor. The type of article was recorded (review, basic science or clinical trial) as well as methodological design (*in vitro* study, animal study, case-report, case series, narrative review/expert opinion, observational study, randomized clinical trial, systematic review/meta-analysis). No exclusion criteria were applied. Meanwhile, to further confirm the citation results, the Scopus™ database was also consulted. For each article, the annual citation rate (ACR) was calculated as the ratio between the number of citations (C) and the number of years (Y) since its publication: ACR = C/Y.

## 5. Conclusions

Within its limitations, this work highlight and confirms the increasing importance of melatonin, which, in perspective, is expected to significantly regulate the rhythm of future research, with predictable new trends going to be related to biomedical and nutritional sciences.

## Figures and Tables

**Table 1:** Top-100 rank of the most cited articles in the field of melatonin research.

Ranking	Article	Citations
1	Valko, M.; Morris, H.; Cronin, M.T.D. Metals, toxicity and oxidative stress. *Curr. Med. Chem.* **2005**, *12*, 1161–1208 [9].	1623
2	Reiter, R.J. Pineal melatonin: Cell biology of its synthesis and of its physiological interactions. *Endocr. Rev.* **1991**, *12*, 151–180 [10].	1572
3	Tan, D.-X.; Chen, L.D.; Poeggeler, B.; Manchester, L.C.; Reiter, R.J.; others. Melatonin: A potent, endogenous hydroxyl radical scavenger. *Endocr. J.* **1993**, *1*, 57–60 [11].	1420
4	Reiter, R.J. The pineal and its hormones in the control of reproduction in mammals. *Endocr. Rev.* **1980**, *1*, 109–131 [12].	1219
5	Lewy, A.J.; Wehr, T.A.; Goodwin, F.K.; Newsome, D.A.; Markey, S.P. Light suppresses melatonin secretion in humans. *Science* **1980**, *210*, 1267–1269 [13].	1105
6	Maritim, A.C.; Sanders, R.A.; Watkins, J.B. Diabetes, oxidative stress, and antioxidants: A review. *J. Biochem. Mol. Toxicol.* **2003**, *17*, 24–38 [14].	975
7	Rodriguez, C.; Mayo, J.C.; Sainz, R.M.; Antolín, I.; Herrera, F.; Martín, V.; Reiter, R.J. Regulation of antioxidant enzymes: A significant role for melatonin. *J. Pineal Res.* **2004**, *36*, 1–9 [15].	886
8	Lerner, A.B.; Case, J.D.; Takahashi, Y.; Lee, T.H.; Mori, W. Isolation of melatonin, the pineal gland factor that lightens melanocytes. *J. Am. Chem. Soc.* **1958**, *80*, 2587–2587 [16].	836
9	Axelrod, J. The pineal gland: A neurochemical transducer. *Science* **1974**, *184*, 1341–1348 [17].	815
10	Brzezinski, A. Melatonin in humans. *N. Engl. J. Med.* **1997**, *336*, 186–195 [18].	802
11	Reppert, S.M.; Weaver, D.R.; Ebisawa, T. Cloning and characterization of a mammalian melatonin receptor that mediates reproductive and circadian responses. *Neuron* **1994**, *13*, 1177–1185 [19].	802
12	Klein, D.C.; Weller, J.L. Indole metabolism in the pineal gland: A circadian rhythm in *N*-acetyltransferase. *Science* **1970**, *169*, 1093–1095 [20].	757
13	Ancoli-Israel, S.; Cole, R.; Alessi, C.; Chambers, M.; Moorcroft, W.; Pollak, C.P. The role of actigraphy in the study of sleep and circadian rhythms. *Sleep* **2003**, *26*, 342–392 [21]	768
14	Tan, D.-X.; Manchester, L.C.; Terron, M.P.; Flores, L.J.; Reiter, R.J. One molecule, many derivatives: A never-ending interaction of melatonin with reactive oxygen and nitrogen species? *J. Pineal Res.* **2007**, *42*, 28–42 [22].	765
15	Toh, K.L.; Jones, C.R.; He, Y.; Eide, E.J.; Hinz, W.A.; Virshup, D.M.; Ptácek, L.J.; Fu, Y.H. An hPer2 phosphorylation site mutation in familial advanced sleep phase syndrome. *Science* **2001**, *291*, 1040–1043 [23].	716
16	Rollag, M.D.; Niswender, G.D. Radioimmunoassay of serum concentrations of melatonin in sheep exposed to different lighting regimens. *Endocrinology* **1976**, *98*, 482–489 [24].	688
17	Reiter, R.J. Oxidative processes and antioxidative defense mechanisms in the aging brain. *FASEB J* **1995**, *9*, 526–533 [25].	678
18	Reiter, R.J. Oxidative damage in the central nervous system: Protection by melatonin. *Prog. Neurobiol.* **1998**, *56*, 359–384 [26].	668
19	Reiter, R.J.; Tan, D.X.; Osuna, C.; Gitto, E. Actions of melatonin in the reduction of oxidative stress. A review. *J. Biomed. Sci.* **2000**, *7*, 444–458 [27].	665
20	Reiter, R.J.; Melchiorri, D.; Sewerynek, E.; Poeggeler, B.; Barlow-Walden, L.; Chuang, J.; Ortiz, G.G.; Acuña-Castroviejo, D. A review of the evidence supporting melatonin’s role as an antioxidant. *J. Pineal Res.* **1995**, *18*, 1–11 [28].	663
21	Reiter, R.J. The melatonin rhythm: Both a clock and a calendar. *Experientia* **1993**, *49*, 654–664 [29].	660
22	Reppert, S.M.; Godson, C.; Mahle, C.D.; Weaver, D.R.; Slaugenhaupt, S.A.; Gusella, J.F. Molecular characterization of a second melatonin receptor expressed in human retina and brain: The Mel1b melatonin receptor. *Proc. Natl. Acad. Sci. USA* **1995**, *92*, 8734–8738 [30].	641
23	Czeisler, C.A.; Duffy, J.F.; Shanahan, T.L.; Brown, E.N.; Mitchell, J.F.; Rimmer, D.W.; Ronda, J.M.; Silva, E.J.; Allan, J.S.; Emens, J.S.; Dijk, D.J.; Kronauer, R.E. Stability, precision, and near-24-hour period of the human circadian pacemaker. *Science* **1999**, *284*, 2177–2181 [31].	607
24	Brainard, G.C.; Hanifin, J.P.; Greeson, J.M.; Byrne, B.; Glickman, G.; Gerner, E.; Rollag, M.D. Action spectrum for melatonin regulation in humans: Evidence for a novel circadian photoreceptor. *J. Neurosci.* **2001**, *21*, 6405–6412 [32].	582
25	Tamarkin, L.; Baird, C.J.; Almeida, O.F. Melatonin: A coordinating signal for mammalian reproduction? *Science* **1985**, *227*, 714–720 [33].	573
26	Lincoln, G.A.; Short, R.V. Seasonal breeding: Nature’s contraceptive. *Recent Prog. Horm. Res.* **1980**, *36*, 1–52 [34].	562
27	Fraser, S.; Cowen, P.; Franklin, M.; Franey, C.; Arendt, J. Direct radioimmunoassay for melatonin in plasma. *Clin. Chem.* **1983**, *29*, 396–397 [35].	554
28	Tan, D.; Reiter, R.J.; Manchester, L.C.; Yan, M.; El-Sawi, M.; Sainz, R.M.; Mayo, J.C.; Kohen, R.; Allegra, M.; Hardeland, R. Chemical and physical properties and potential mechanisms: melatonin as a broad spectrum antioxidant and free radical scavenger. *Curr. Top. Med. Chem.* **2002**, *2*, 181–197 [36].	539
29	Karsch, F.J.; Bittman, E.L.; Foster, D.L.; Goodman, R.L.; Legan, S.J.; Robinson, J.E. Neuroendocrine basis of seasonal reproduction. *Recent Prog. Horm. Res.* **1984**, *40*, 185–232 [37].	527
30	Cao, G.; Prior, R.L. Comparison of different analytical methods for assessing total antioxidant capacity of human serum. *Clin. Chem.* **1998**, *44*, 1309–1315 [38].	516
31	Provencio, I.; Rodriguez, I.R.; Jiang, G.; Hayes, W.P.; Moreira, E.F.; Rollag, M.D. A novel human opsin in the inner retina. *J. Neurosci.* **2000**, *20*, 600–605 [39].	514
32	Schernhammer, E.S.; Laden, F.; Speizer, F.E.; Willett, W.C.; Hunter, D.J.; Kawachi, I.; Colditz, G.A. Rotating night shifts and risk of breast cancer in women participating in the nurses’ health study. *J. Natl. Cancer Inst.* **2001**, *93*, 1563–1568 [40].	514
33	Lewy, A.J.; Sack, R.L.; Miller, L.S.; Hoban, T.M. Antidepressant and circadian phase-shifting effects of light. *Science* **1987**, *235*, 352–354 [41].	511
34	Morgan, P.J.; Barrett, P.; Howell, H.E.; Helliwell, R. Melatonin receptors: Localization, molecular pharmacology and physiological significance. *Neurochem. Int.* **1994**, *24*, 101–146 [42].	498
35	Kidd, P. Th1/Th2 balance: the hypothesis, its limitations, and implications for health and disease. *Altern. Med. Rev. J. Clin. Ther.* **2003**, *8*, 223–246 [43].	495
36	Sun, Z.S.; Albrecht, U.; Zhuchenko, O.; Bailey, J.; Eichele, G.; Lee, C.C. RIGUI, a putative mammalian ortholog of the Drosophila period gene. *Cell* **1997**, *90*, 1003–1011 [44].	494
37	Tosini, G.; Menaker, M. Circadian rhythms in cultured mammalian retina. *Science* **1996**, *272*, 419–421 [45].	488
38	Allegra, M.; Reiter, R.J.; Tan, D.-X.; Gentile, C.; Tesoriere, L.; Livrea, M.A. The chemistry of melatonin’s interaction with reactive species. *J. Pineal Res.* **2003**, *34*, 1–10 [46].	479
39	Ascher, J.A.; Cole, J.O.; Colin, J.N.; Feighner, J.P.; Ferris, R.M.; Fibiger, H.C.; Golden, R.N.; Martin, P.; Potter, W.Z.; Richelson, E. Bupropion: A review of its mechanism of antidepressant activity. *J. Clin. Psychiatry* **1995**, *56*, 395–401 [47].	478
40	Lewy, A.J.; Ahmed, S.; Jackson, J.M.; Sack, R.L. Melatonin shifts human circadian rhythms according to a phase-response curve. *Chronobiol. Int.* **1992**, *9*, 380–392 [48]	464
41	Tamarkin, L.; Westrom, W.K.; Hamill, A.I.; Goldman, B.D. Effect of melatonin on the reproductive systems of male and female Syrian hamsters: A diurnal rhythm in sensitivity to melatonin. *Endocrinology* **1976**, *99*, 1534–1541 [49].	457
42	Liu, C.; Weaver, D.R.; Jin, X.; Shearman, L.P.; Pieschl, R.L.; Gribkoff, V.K.; Reppert, S.M. Molecular dissection of two distinct actions of melatonin on the suprachiasmatic circadian clock. *Neuron* **1997**, *19*, 91–102 [50].	446
43	Redman, J.; Armstrong, S.; Ng, K.T. Free-running activity rhythms in the rat: Entrainment by melatonin. *Science* **1983**, *219*, 1089–1091 [51].	441
44	Maestroni, G.J. The immunoneuroendocrine role of melatonin. *J. Pineal Res.* **1993**, *14*, 1–10 [52].	439
45	Dollins, A.B.; Zhdanova, I.V.; Wurtman, R.J.; Lynch, H.J.; Deng, M.H. Effect of inducing nocturnal serum melatonin concentrations in daytime on sleep, mood, body temperature, and performance. *Proc. Natl. Acad. Sci. USA* **1994**, *91*, 1824–1828 [53].	432
46	Thapan, K.; Arendt, J.; Skene, D.J. An action spectrum for melatonin suppression: evidence for a novel non-rod, non-cone photoreceptor system in humans. *J. Physiol.* **2001**, *535*, 261–267 [54].	431
47	Nicholls, T.J.; Goldsmith, A.R.; Dawson, A. Photorefractoriness in birds and comparison with mammals. *Physiol. Rev.* **1988**, *68*, 133–176 [55].	427
48	Goldman, B.D. Mammalian photoperiodic system: Formal properties and neuroendocrine mechanisms of photoperiodic time measurement. *J. Biol. Rhythms* **2001**, *16*, 283–301 [56].	425
49	Davis, S.; Mirick, D.K.; Stevens, R.G. Night shift work, light at night, and risk of breast cancer. *J. Natl. Cancer Inst.* **2001**, *93*, 1557–1562 [57].	422
50	Reiter, R.; Tang, L.; Garcia, J.J.; Muñoz-Hoyos, A. Pharmacological actions of melatonin in oxygen radical pathophysiology. *Life Sci.* **1997**, *60*, 2255–2271 [58].	422
51	Axelrod, J.; Wurtman, R.J.; Snyder, S.H. Control of hydroxyindole o-methyltransferase activity in the rat pineal gland by environmental lighting. *J. Biol. Chem.* **1965**, *240*, 949–954 [59].	420
52	Grohmann, U.; Fallarino, F.; Puccetti, P. Tolerance, DCs and tryptophan: Much ado about IDO. *Trends Immunol.* **2003**, *24*, 242–248 [60].	419
53	Reiter, R.J.; Tan, D.X.; Manchester, L.C.; Qi, W. Biochemical reactivity of melatonin with reactive oxygen and nitrogen species: a review of the evidence. *Cell Biochem. Biophys.* **2001**, *34*, 237–256 [61].	418
54	Barlow-Walden, L.R.; Reiter, R.J.; Abe, M.; Pablos, M.; Menendez-Pelaez, A.; Chen, L.D.; Poeggeler, B. Melatonin stimulates brain glutathione peroxidase activity. *Neurochem. Int.* **1995**, *26*, 497–502 [62].	415
55	Pieri, C.; Marra, M.; Moroni, F.; Recchioni, R.; Marcheselli, F. Melatonin: A peroxyl radical scavenger more effective than vitamin E. *Life Sci.* **1994**, *55*, PL271–276 [63].	414
56	Reppert, S.M.; Weaver, D.R.; Rivkees, S.A.; Stopa, E.G. Putative melatonin receptors in a human biological clock. *Science* **1988**, *242*, 78–81 [64].	413
57	Kamberi, I.A.; Mical, R.S.; Porter, J.C. Effects of melatonin and serotonin on the release of FSH and prolactin. *Endocrinology* **1971**, *88*, 1288–1293 [65].	409
58	Pardridge, W.M. Transport of protein-bound hormones into tissues *in vivo*. *Endocr. Rev.* **1981**, *2*, 103–123 [66].	407
59	Sugden, D.; Vanecek, J.; Klein, D.C.; *et al.* Activation of protein kinase C potentiates isoprenaline-induced cyclic AMP accumulation in rat pinealocytes. *Nature* **1985**, *314*, 359–361 [67].	406
60	Dawson, A.; King, V.M.; Bentley, G.E.; Ball, G.F. Photoperiodic control of seasonality in birds. *J. Biol. Rhythms* **2001**, *16*, 365–380 [68].	401
61	Dubocovich, M.L. Melatonin is a potent modulator of dopamine release in the retina. *Nature* **1983**, *306*, 782–784 [69].	399
62	Wurtman, R.J.; Axelrod, J.; Phillips, L.S. Melatonin synthesis in the pineal gland: control by light. *Science* **1963**, *142*, 1071–1073 [70].	392
63	Bartness, T.J.; Powers, J.B.; Hastings, M.H.; Bittman, E.L.; Goldman, B.D. The timed infusion paradigm for melatonin delivery: What has it taught us about the melatonin signal, its reception, and the photoperiodic control of seasonal responses? *J. Pineal Res.* **1993**, *15*, 161–190 [71].	394
64	Klein, D.C.; Coon, S.L.; Roseboom, P.H.; Weller, J.L.; Bernard, M.; Gastel, J.A.; Zatz, M.; Iuvone, P.M.; Rodriguez, I.R.; Bégay, V.; *et al*. The melatonin rhythm-generating enzyme: molecular regulation of serotonin *N*-acetyltransferase in the pineal gland. *Recent Prog. Horm. Res.* **1997**, *52*, 307–357; discussion 357–358 [72].	391
65	Vanecek, J. Cellular mechanisms of melatonin action. *Physiol. Rev.* **1998**, *78*, 687–721 [73]	389
66	Carter, D.S.; Goldman, B.D. Antigonadal effects of timed melatonin infusion in pinealectomized male Djungarian hamsters (Phodopus sungorus sungorus): Duration is the critical parameter. *Endocrinology* **1983**, *113*, 1261–1267 [74].	385
67	Zeitzer, J.M.; Dijk, D.J.; Kronauer, R.; Brown, E.; Czeisler, C. Sensitivity of the human circadian pacemaker to nocturnal light: Melatonin phase resetting and suppression. *J. Physiol.* **2000**, *526* (Pt 3), 695–702 [75].	385
68	Vanĕcek, J.; Pavlík, A.; Illnerová, H. Hypothalamic melatonin receptor sites revealed by autoradiography. *Brain Res.* **1987**, *435*, 359–362 [76].	368
69	Hill, S.M.; Blask, D.E. Effects of the pineal hormone melatonin on the proliferation and morphological characteristics of human breast cancer cells (MCF-7) in culture. *Cancer Res.* **1988**, *48*, 6121–6126 [77].	363
70	Kamberi, I.A.; Mical, R.S.; Porter, J.C. Effect of anterior pituitary perfusion and intraventricular injection of catecholamines and indoleamines on LH release. *Endocrinology* **1970**, *87*, 1–12 [78].	360
71	Pandi-Perumal, S.R.; Srinivasan, V.; Maestroni, G.J. M.; Cardinali, D.P.; Poeggeler, B.; Hardeland, R. Melatonin: Nature’s most versatile biological signal? *FEBS J.* **2006**, *273*, 2813–2838 [79].	376
72	Galano, A.; Tan, D.X.; Reiter, R.J. Melatonin as a natural ally against oxidative stress: A physicochemical examination. *J. Pineal Res.* **2011**, *51*, 1–16 [80].	399
73	Bromage, N.; Porter, M.; Randall, C. The environmental regulation of maturation in farmed finfish with special reference to the role of photoperiod and melatonin. *Aquaculture* **2001**, *197*, 63–98 [81].	368
74	Poeggeler, B.; Reiter, R.J.; Tan, D.X.; Chen, L.D.; Manchester, L.C. Melatonin, hydroxyl radical-mediated oxidative damage, and aging: a hypothesis. *J. Pineal Res.* **1993**, *14*, 151–168 [82].	362
75	Jezek, P.; Hlavatá, L. Mitochondria in homeostasis of reactive oxygen species in cell, tissues, and organism. *Int. J. Biochem. Cell Biol.* **2005**, *37*, 2478–2503 [83].	356
76	Ebisawa, T.; Karne, S.; Lerner, M.R.; Reppert, S.M. Expression cloning of a high-affinity melatonin receptor from Xenopus dermal melanophores. *Proc. Natl. Acad. Sci. USA* **1994**, *91*, 6133–6137 [84].	353
77	Stehle, J.H.; Foulkes, N.S.; Molina, C.A.; Simonneaux, V.; Pévet, P.; Sassone-Corsi, P. Adrenergic signals direct rhythmic expression of transcriptional repressor CREM in the pineal gland. *Nature* **1993**, *365*, 314–320 [85].	353
78	Dubocovich, M.L. Melatonin receptors: are there multiple subtypes? *Trends Pharmacol. Sci.* **1995**, *16*, 50–56 [86].	350
79	Antolín, I.; Rodríguez, C.; Saínz, R.M.; Mayo, J.C.; Uría, H.; Kotler, M.L.; Rodríguez-Colunga, M.J.; Tolivia, D.; Menéndez-Peláez, A. Neurohormone melatonin prevents cell damage: effect on gene expression for antioxidant enzymes. *FASEB J.* **1996**, *10*, 882–890 [87].	349
80	Cassone, V.M. Effects of melatonin on vertebrate circadian systems. *Trends Neurosci.* **1990**, *13*, 457–464 [88].	346
81	Prokopenko, I.; Langenberg, C.; Florez, J.C.; Saxena, R.; Soranzo, N.; Thorleifsson, G.; Loos, R.J.F.; Manning, A.K.; Jackson, A.U.; Aulchenko, Y.; *et al.* Variants in MTNR1B influence fasting glucose levels. *Nat. Genet.* **2009**, *41*, 77–81 [89].	346
82	Czeisler, C.A.; Shanahan, T.L.; Klerman, E.B.; Martens, H.; Brotman, D.J.; Emens, J.S.; Klein, T.; Rizzo, J.F. Suppression of melatonin secretion in some blind patients by exposure to bright light. *N. Engl. J. Med.* **1995**, *332*, 6–11 [90].	345
83	Walther, D.J.; Bader, M. A unique central tryptophan hydroxylase isoform. *Biochem. Pharmacol.* **2003**, *66*, 1673–1680 [91].	345
84	Suntres, Z.E. Role of antioxidants in paraquat toxicity. *Toxicology* **2002**, *180*, 65–77 [92].	344
85	Nemecek, G.M.; Coughlin, S.R.; Handley, D.A.; Moskowitz, M.A. Stimulation of aortic smooth muscle cell mitogenesis by serotonin. *Proc. Natl. Acad. Sci. USA* **1986**, *83*, 674–678 [93].	338
86	Schernhammer, E.S.; Laden, F.; Speizer, F.E.; Willett, W.C.; Hunter, D.J.; Kawachi, I.; Fuchs, C.S.; Colditz, G.A. Night-shift work and risk of colorectal cancer in the nurses’ health study. *J. Natl. Cancer Inst.* **2003**, *95*, 825–828 [94].	338
87	Ruby, N.F.; Brennan, T.J.; Xie, X.; Cao, V.; Franken, P.; Heller, H.C.; O’Hara, B.F. Role of melanopsin in circadian responses to light. *Science* **2002**, *298*, 2211–2213 [95].	328
88	Simonneaux, V.; Ribelayga, C. Generation of the melatonin endocrine message in mammals: A review of the complex regulation of melatonin synthesis by norepinephrine, peptides, and other pineal transmitters. *Pharmacol. Rev.* **2003**, *55*, 325–395 [96].	326
89	Reppert, S.M.; Weaver, D.R.; Godson, C. Melatonin receptors step into the light: cloning and classification of subtypes. *Trends Pharmacol. Sci.* **1996**, *17*, 100–102 [97].	325
90	Wurtman, R.J.; Axelrod, J.; Chu, E.W. Melatonin, a Pineal Substance: Effect on the Rat Ovary. *Science* **1963**, *141*, 277–278 [98].	324
91	Kopin, I.J.; Pare, C.M.; Axelrod, J.; Weissbach, H. The fate of melatonin in animals. *J. Biol. Chem.* **1961**, *236*, 3072–3075 [99].	323
92	Bubenik, G.A. Gastrointestinal melatonin: Localization, function, and clinical relevance. *Dig. Dis. Sci.* **2002**, *47*, 2336–2348 [100].	323
93	Lerner, A.B.; Case, J.D.; Heinzelman, R.V. Structure of melatonin. *J. Am. Chem. Soc.* **1959**, *81*, 6084–6085 [101].	320
94	Hoffmann, K. The influence of photoperiod and melatonin on testis size, body weight, and pelage colour in the Djungarian hamster (*Phodopus sungorus*). *J. Comp. Physiol.* **1973**, *85*, 267–282 [102].	316
95	Maestroni, G.J.; Conti, A.; Pierpaoli, W. Role of the pineal gland in immunity. Circadian synthesis and release of melatonin modulates the antibody response and antagonizes the immunosuppressive effect of corticosterone. *J. Neuroimmunol.* **1986**, *13*, 19–30 [103].	314
96	Garfinkel, D.; Laudon, M.; Nof, D.; Zisapel, N. Improvement of sleep quality in elderly people by controlled-release melatonin. *Lancet Lond. Engl.* **1995**, *346*, 541–544 [104].	314
97	Nelson, R.J.; Demas, G.E. Seasonal changes in immune function. *Q. Rev. Biol.* **1996**, *71*, 511–548 [105].	314
98	Weaver, D.R.; Rivkees, S.A.; Reppert, S.M. Localization and characterization of melatonin receptors in rodent brain by in vitro autoradiography. *J. Neurosci.* **1989**, *9*, 2581–2590 [106].	312
99	Tan, D.X.; Manchester, L.C.; Reiter, R.J.; Qi, W.B.; Karbownik, M.; Calvo, J.R. Significance of melatonin in antioxidative defense system: reactions and products. *Biol. Signals Recept.* **2000**, *9*, 137–159 [107].	310
100	Davidse, L.C.; Flach, W. Differential binding of methyl benzimidazol-2-yl carbamate to fungal tubulin as a mechanism of resistance to this antimitotic agent in mutant strains of *Aspergillus nidulans. J. Cell Biol.* **1977**, *72*, 174–193 [108].	309

**Table 2:** Top 10 articles according to their annual citation rate (ACR, citations/year).

Ranking	Article	ACR
1	Valko, M.; Morris, H.; Cronin, M.T. D. Metals, toxicity and oxidative stress. *Curr. Med. Chem.* **2005**, *12*, 1161–1208 [9].	162.3
2	Galano, A.; Tan, D.X.; Reiter, R.J. Melatonin as a natural ally against oxidative stress: A physicochemical examination. *J. Pineal Res.* **2011**, *51*, 1–16 [80].	99.7
3	Tan, D.-X.; Manchester, L.C.; Terron, M.P.; Flores, L.J.; Reiter, R.J. One molecule, many derivatives: A never-ending interaction of melatonin with reactive oxygen and nitrogen species? *J. Pineal Res.* **2007**, *42*, 28–42 [22].	95.6
4	Maritim, A.C.; Sanders, R.A.; Watkins, J.B. Diabetes, oxidative stress, and antioxidants: A review. *J. Biochem. Mol. Toxicol.* **2003**, *17*, 24–38 [14].	81.25
5	Rodriguez, C.; Mayo, J.C.; Sainz, R.M.; Antolín, I.; Herrera, F.; Martín, V.; Reiter, R.J. Regulation of antioxidant enzymes: A significant role for melatonin. *J. Pineal Res.* **2004**, *36*, 1–9 [15].	80.5
6	Reiter, R.J. Pineal melatonin: Cell biology of its synthesis and of its physiological interactions. *Endocr. Rev.* **1991**, *12*, 151–180 [10].	65.5
7	Tan, D.-X.; Chen, L.D.; Poeggeler, B.; Manchester, L.C.; Reiter, R.J.; others. Melatonin: A potent, endogenous hydroxyl radical scavenger. *Endocr. J.* **1993**, *1*, 57–60 [11].	64.5
8	Ancoli-Israel, S.; Cole, R.; Alessi, C.; Chambers, M.; Moorcroft, W.; Pollak, C.P. The role of actigraphy in the study of sleep and circadian rhythms. *Sleep* **2003**, *26*, 342–392 [21].	64.0
9	Prokopenko, I.; Langenberg, C.; Florez, J.C.; Saxena, R.; Soranzo, N.; Thorleifsson, G.; Loos, R.J.F.; Manning, A.K.; Jackson, A.U.; Aulchenko, Y.; Potter, S.C.; *et al*. Variants in MTNR1B influence fasting glucose levels. *Nat. Genet.* **2009**, *41*, 77–81 [89].	57.6
10	Toh, K.L.; Jones, C.R.; He, Y.; Eide, E.J.; Hinz, W.A.; Virshup, D.M.; Ptácek, L.J.; Fu, Y.H. An hPer2 phosphorylation site mutation in familial advanced sleep phase syndrome. *Science* **2001**, *291*, 1040–1043 [23].	51.1

**Table 3:** The top 10 most cited reviews.

Ranking	Article	Citations
1	Valko, M.; Morris, H.; Cronin, M.T. D. Metals, toxicity and oxidative stress. *Curr. Med. Chem.* **2005**, *12*, 1161–1208 [9].	1623
2	Reiter, R.J. Pineal melatonin: Cell biology of its synthesis and of its physiological interactions. *Endocr. Rev.* **1991**, *12*, 151–180 [10].	1572
3	Reiter, R.J. The pineal and its hormones in the control of reproduction in mammals. *Endocr. Rev.* **1980**, *1*, 109–131 [12].	1219
4	Maritim, A.C.; Sanders, R.A.; Watkins, J.B. Diabetes, oxidative stress, and antioxidants: A review. *J. Biochem. Mol. Toxicol.* **2003**, *17*, 24–38 [14].	975
5	Rodriguez, C.; Mayo, J.C.; Sainz, R.M.; Antolín, I.; Herrera, F.; Martín, V.; Reiter, R.J. Regulation of antioxidant enzymes: A significant role for melatonin. *J. Pineal Res.* **2004**, *36*, 1–9 [15].	886
6	Lerner A.B.; Case, J.D.; Takahashi, Y.; Lee, T.; Mori, W. Isolation of melatonin, the pineal gland factor that lightens melanocytes. *J. Am. Chem. Soc.* **1958**, *80*, 2587–2587 [16].	836
7	Axelrod, J. The pineal gland: A neurochemical transducer. *Science* **1974**, *184*, 1341–1348 [17].	815
8	Brzezinski, A. Melatonin in humans. *N. Engl. J. Med.* **1997**, *336*, 186–195 [18].	802
9	Ancoli-Israel, S.; Cole, R.; Alessi, C.; Chambers, M.; Moorcroft, W.; Pollak, C.P. The role of actigraphy in the study of sleep and circadian rhythms. *Sleep* **2003**, *26*, 342–392 [21].	768
10	Klein, D.C.; Weller, J.L. Indole metabolism in the pineal gland: A circadian rhythm in *N*-acetyltransferase. *Science* **1970**, *169*, 1093–1095 [20].	757

**Table 4:** The top 10 most cited original articles.

Ranking	Article	Citations
1	Tan, D.-X.; Chen, L.D.; Poeggeler, B.; Manchester, L.C.; Reiter, R.J.; others. Melatonin: A potent, endogenous hydroxyl radical scavenger. *Endocr J* **1993**, *1*, 57–60 [11].	1420
2	Lewy, A.J.; Wehr, T.A.; Goodwin, F.K.; Newsome, D.A.; Markey, S.P. Light suppresses melatonin secretion in humans. *Science* **1980**, *210*, 1267–1269 [13].	1105
3	Reppert, S.M.; Weaver, D.R.; Ebisawa, T. Cloning and characterization of a mammalian melatonin receptor that mediates reproductive and circadian responses. *Neuron* **1994**, *13*, 1177–1185 [19].	802
4	Toh, K.L.; Jones, C.R.; He, Y.; Eide, E.J.; Hinz, W.A.; Virshup, D.M.; Ptácek, L.J.; Fu, Y.H. An hPer2 phosphorylation site mutation in familial advanced sleep phase syndrome. *Science* **2001**, *291*, 1040–1043 [23].	716
5	Rollag, M.D.; Niswender, G.D. Radioimmunoassay of serum concentrations of melatonin in sheep exposed to different lighting regimens. *Endocrinology* **1976**, *98*, 482–489 [24].	688
6	Reppert, S.M.; Godson, C.; Mahle, C.D.; Weaver, D.R.; Slaugenhaupt, S.A.; Gusella, J.F. Molecular characterization of a second melatonin receptor expressed in human retina and brain: The Mel1b melatonin receptor. *Proc. Natl. Acad. Sci. USA* **1995**, *92*, 8734–8738 [30].	641
7	Fraser, S.; Cowen, P.; Franklin, M.; Franey, C.; Arendt, J. Direct radioimmunoassay for melatonin in plasma. *Clin. Chem.* **1983**, *29*, 396–397 [35].	554
8	Cao, G.; Prior, R.L. Comparison of different analytical methods for assessing total antioxidant capacity of human serum. *Clin. Chem.* **1998**, *44*, 1309–1315 [38].	516
9	Provencio, I.; Rodriguez, I.R.; Jiang, G.; Hayes, W.P.; Moreira, E.F.; Rollag, M.D. A novel human opsin in the inner retina. *J. Neurosci. Off. J. Soc. Neurosci.* **2000**, *20*, 600–605 [39].	514
10	Schernhammer, E.S.; Laden, F.; Speizer, F.E.; Willett, W.C.; Hunter, D.J.; Kawachi, I.; Colditz, G.A. Rotating night shifts and risk of breast cancer in women participating in the nurses’ health study. *J. Natl. Cancer Inst.* **2001**, *93*, 1563–1568 [40].	514

**Table 5:** Ranking of the journals with articles within the top 100 list.

Ranking	Journal	No Articles
1	Science (33.611)	13
2	Journal of Pineal Research (9.600)	8
3	Endocrinology (4.503)	5
4	Proceedings of the National Academy of Sciences USA (9.674)	4
5	Endocrine Reviews (21.059)	3
6	Journal of Neuroscience (6.344)	3
7	Journal of the National Cancer Institute (12.583)	3
8	Nature (41.456)	3
9	Recent Progress in Hormone Research	3
10	Clinical Chemistry (7.911)	2
11	FASEB Journal (5.043)	2
12	Journal of Biological Rhythms (4.573)	2
13	Journal of Physiology - London (5.037)	2
14	Journal of the American Chemical Society (12.113)	2
15	Life Sciences (2.702)	2
16	Neurochemistry International (3.092)	2
17	Neuron (15.054)	2
18	Physiological Reviews (27.324)	2
19	The Journal of Biological Chemistry (4.573)	2
20	The New England Journal of Medicine (55.873)	2
21	Trends in Pharmacological Sciences (11.539)	2
22	Aquaculture (1.878)	1
23	Alternative Medicine Review (3.833)	1
24	Biochemical Pharmacology (5.009)	1
25	Biological Signals and Receptors (2.000)	1
26	Brain Research (2.843)	1
27	Cancer Research (9.329)	1
28	Cell (32.242)	1
29	Cell Biochemistry and Biophysics (1.680)	1
30	Chronobiology International (3.343)	1
31	Current Medicinal Chemistry (3.853)	1
32	Current Topics in Medicinal Chemistry (3.402)	1
33	Digestive Diseases and Sciences (2.613)	1
34	Endocrine Journal (1.997)	1
35	Experientia (5.808)	1
36	FEBS Journal (4.001)	1
37	International Journal of Biochemistry and Cell Biology (4.046)	1
38	Journal of Biochemistry and Molecular Toxicology (1.925)	1
39	Journal of Biomedical Science (2.763)	1
40	Journal of Clinical Psychiatry (5.498)	1
41	Journal of Comparative Physiology (2.036)	1
42	Journal of Neuroimmunology (2.467)	1
43	Nature Genetics (29.352)	1
44	Pharmacological Reviews (17.099)	1
45	Progress in Neurobiology (9.992)	1
46	Quarterly Review of Biology (4.889)	1
47	Sleep (4.591)	1
48	The Journal of Cell Biology (9.834)	1
49	The Lancet (45.217)	1
50	Toxicology (3.621)	1
51	Trends in Immunology (10.399)	1
52	Trends in Neurosciences (13.555)	1
	**Total**	**100**

**Table 6:** Top five authors with the most cited papers on melatonin research.

Name	First Author	Co-Author	Last Author	Total
1. Reiter, RJ	9	4	3	16
2. Tan, D-X	4	3	2	9
3. Reppert, SM	4		1	5
4. Weaver, DR	1	4	0	5
5. Axelrod, J	2	2	1	5
6. Manchester, LC	0	2	3	5

## Abbreviations

The following abbreviations are used in this manuscript:
Annual Citation Rate (ACR)
Journal of Citation Report (JCR)
Institute for Scientific Information (ISI)
